# Notch signalling in cancer progression and bone metastasis

**DOI:** 10.1038/bjc.2011.497

**Published:** 2011-11-10

**Authors:** N Sethi, Y Kang

**Affiliations:** 1 Department of Molecular Biology, Princeton University, Washington Road, LTL 255, Princeton, NJ 08544, USA; 2 Robert Wood Johnson Medical School, Piscataway, NJ 08544, USA; 3 Genomic Instability and Tumor Progression Program, Cancer Institute of New Jersey, New Brunswick, NJ 08903, USA

**Keywords:** Notch signalling, bone metastasis, Jagged1, tumour progression

## Abstract

Classically known for its indispensible role in embryonic development, the Notch signalling pathway is gaining recognition for its regulation of adult tissue homoeostasis and aberrant activation in disease pathogenesis. The pathway has been implicated in cancer initiation and development, as well as early stages of cancer progression by regulating conserved cellular programs such as the epithelial-to-mesenchymal transition. We recently extended the role of Notch signalling to late stages of tumour progression by elucidating a stroma-dependent mechanism for the pathway in osteolytic bone metastasis. Of clinical significance, disrupting the Notch pathway and associated molecular mediators of Notch-dependent bone metastasis may provide novel therapeutic strategies to combat aggressive bone metastatic disease.

## The Notch signalling pathway

The Notch pathway regulates cell fate decisions during embryonic development by facilitating short-range signalling between neighbouring cells that are in physical contact. Initially discovered for yielding a ‘notched’ wing phenotype in *Drosophila* due to a partial loss of function, the pathway has since been implicated in specifying the development of several different tissues and organisms (Artavanis-Tsakonas *et al*, 1999). In a context-dependent manner, Notch signalling coordinates a wide range of fundamental processes and cellular programs including proliferation, apoptosis, migration, growth, and differentiation. The pathway has only recently been associated with the maintenance of adult tissue and pathogenesis of cancer.

The four mammalian Notch receptors are single-pass type-1 transmembrane proteins that are expressed on the cell surface (Logeat *et al*, 1998). The extracellular domain contains epidermal growth factor (EGF)-like repeats that are responsible for ligand binding and a negative regulatory region that prevents receptor activation in the absence of ligands. The intracellular domain of Notch receptors contain three common regions: (1) a subtransmembrane region that associates with transcriptional components in the nucleus (Honjo, 1996), (2) six tandem ankyrin repeats that are necessary for transcriptional activity, and (3) a PEST sequence that regulates stability and protein turnover (Greenwald, 1994; Bray, 2006). The receptors are activated via interactions with Notch ligands, which are also type-I transmembrane proteins with multiple EGF-like repeats in their extracellular domain (D’Souza *et al*, 2008). The five mammalian Notch ligands are separated into two subgroups: Delta-like (Dll1, Dll3, and Dll4) or Serrate-like (Jagged1 and Jagged2), based on the structural similarity with their *Drosophila* homologues (Bray, 2006). Ultimately, the diverse functions affected by the Notch pathway are dependent on the signalling interaction between neighbouring cells (Sprinzak *et al*, 2010).

**Figure 1:**
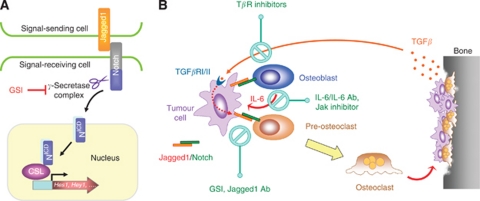
Jagged1/Notch signalling in bone metastasis. (**A**) Schematic representation of Jagged1/Notch mediated juxtacrine signalling between neighbouring cells. The Jagged1 ligand expressed by the signal-sending cell is recognised by the Notch family receptors expressed by the signal-receiving cells and initiates a series of proteolytic cleavage events that liberates the intracellular domain of the Notch receptor (N^ICD^). N^ICD^ interacts with the DNA-binding protein CSL to turn it from a transcriptional repressor to an activator. *γ*-secretase inhibitor (GSI) blocks intracellular cleavage of Notch and inhibits Notch signalling. (**B**) Tumour-derived Jagged1 promotes osteolytic bone metastasis of breast cancer. Jagged1 is overexpressed in breast tumour cells that are highly metastatic to bone and engages bone stromal cells by activating Notch signalling in these cells. Notch signalling promotes the expression and secretion of IL-6, which feeds back to tumour cells to stimulate growth and resistance to chemotherapy. Meanwhile, Notch signalling directly activates osteoclast maturation, thereby exacerbating osteolytic bone metastasis. The destruction of bone matrix releases abundant amount of TGF-*β*, which further activates the expression of Jagged1 in tumour cells through a Smad-dependent fashion. The findings indicate the therapeutic potential of reducing osteolytic bone metastasis by targeting Notch, TGF-*β* and IL-6 through different classes of inhibitors. Figure adopted from Sethi *et al* (2011).

The Notch pathway is activated when a signal-sending cell expressing a membrane-bound ligand physically interacts with a signal-receiving cell expressing a Notch receptor (Gray *et al*, 1999). Upon ligand binding, the Notch receptor is cleaved twice, first by an extracellular matrix metalloprotease (Brou *et al*, 2000) and then by the transmembrane protease complex γ-secretase, releasing the Notch intracellular domain (Schroeter *et al*, 1998; Mumm *et al*, 2000; Six *et al*, 2003) (Figure 1A). After dissociating from the cell membrane, the Notch intracellular domain translocates to the nucleus where it interacts with the DNA-binding protein CSL (Rbp-Jκ in mice; CBF1 in humans) to affect transcriptional responses (Jarriault *et al*, 1995; Wu *et al*, 2000). In a recent example, genomic analysis using Chip–chip arrays demonstrated that the promoter region of Notch1-regulated genes were bound by CSL, which helped reveal several new CSL-dependent targets of Notch signalling (Hamidi *et al*, 2011). The most prominent targets of the Notch pathway include a set of basic helix–loop–helix factors of the hairy and enhancer of split (Hes) and Hes-related repressor protein (Hey) families (Iso *et al*, 2003; Kopan and Ilagan, 2009). These transcription factors execute Notch signalling functions, including maintenance of stem cells, specification of cell fate, differentiation, proliferation, and apoptosis (Radtke and Raj, 2003). Although many of the essential pathway components have been identified, we are still uncovering new regulators of Notch signalling using fundamental developmental and molecular biology (Wang *et al*, 2008; Karaczyn *et al*, 2010; Sethi *et al*, 2010; Dalton *et al*, 2011).

## Notch signalling and cancer

An oncogenic role for Notch was first demonstrated in T-cell acute lymphoblastic leukaemia (T-ALL) with the identification of a chromosomal translocation that resulted in the expression of a truncated, constitutively active form of the Notch1 protein in T-cells (Ellisen *et al*, 1991). Experimental work using transgenic mouse models revealed that constitutive activity of the Notch pathway in hematopoietic progenitors drives the specification of an immature T-cell lineage, which then evolves into a highly aggressive monoclonal T-cell leukaemia with the cooperation of additional mutations (Pear *et al*, 1996; Radtke *et al*, 1999). Further clinical evaluation revealed activating mutations of *Notch1* in more than 50% of patient cases (Weng *et al*, 2004). The characterisation of aberrant Notch signalling in T-cell leukaemia provided a foundation for researchers to explore the pathway's importance in other cancers (Table 1).

The Notch pathway has been implicated in the tumourigenesis of several solid tumour malignancies including, but not limited to, non-small cell lung adenocarcinoma (Dang *et al*, 2000), melanoma (Bedogni *et al*, 2008), ovarian carcinoma (Park *et al*, 2006), medulloblastoma (Hallahan *et al*, 2004), and Kaposi's sarcoma (Curry *et al*, 2005). The pathway has also been shown to contribute to the pathogenesis of breast cancer. Constitutive activation of the Notch pathway in mammary tissue leads to the development of breast cancer in distinct mouse models. These studies employed the mouse mammary tumour virus (MMTV) system, which led to the identification of the *Notch1* and *Notch4* gene loci as frequent viral insertion sites that consequently resulted in the development of breast adenocarcinoma (Gallahan and Callahan, 1987). Similar to the mechanism in T-ALL, the MMTV insertion resulted in the constitutive expression of an active truncated form of the Notch receptor, revealing an oncogenic role for Notch signalling in mammary tissue (Jhappan *et al*, 1992; Dievart *et al*, 1999). Several studies have since shown that constitutive activation of the pathway confers tumourigenic properties to mammary epithelial cells (Raafat *et al*, 2004; Zang *et al*, 2007). However, unlike T-ALL, it is ambiguous as to how the pathway is aberrantly activated in human solid tumour malignancy, as underlying genomic mutations are rarely found in patient samples. Considering the lack of evidence showing genetic mutations in *Notch*, there should be greater emphasis on uncovering the source of Notch signalling activation in solid tumours. One avenue of exploration is characterising the potential heterogeneity of ligand and receptor expression within the primary tumour. Along this line of reasoning, a possible mechanism would involve a subset of primary tumour cells expressing Notch ligands, which have been shown to be aberrantly induced by local microenvironment paracine signals (Zeng *et al*, 2005), may activate the Notch pathway in a different subset of primary tumour, or signal-receiving, cells. Such a scenario would parallel Notch signalling events that take place during development when executing cell fate decisions (e.g. lateral specification) (Heitzler and Simpson, 1991; Greenwald, 1998; Sassoli *et al*, 2011).

Beyond its role in tumourigenesis, Notch signalling has also been associated with cancer progression, particularly through its regulation of the epithelial-to-mesenchymal transition (EMT). Epithelial-to-mesenchymal transition is a conserved cellular programme that confers mesenchymal features to epithelial cells during development and is postulated to provide epithelial tumour cells with migratory properties, facilitating early steps in metastasis such as invasion. The Notch pathway regulates EMT with the contribution of several players and processes, such as TGF*β* signalling (Timmerman *et al*, 2004; Zavadil *et al*, 2004), *β*-catenin activity (Balint *et al*, 2005), the transcription factor Slug (Leong *et al*, 2007), and most recently hypoxia (Bedogni *et al*, 2008; Sahlgren *et al*, 2008). Notch signalling has also shown to directly regulate mediators of invasion, such as matrix metalloproteinase-9 and vascular endothelial growth factor (Wang *et al*, 2006). Interestingly, the Notch ligand Jagged1 is also associated with cancer progression; analysis of clinical samples revealed that it is highly expressed in metastatic prostate cancer compared with localised disease (Santagata *et al*, 2004) and overexpressed in breast cancer patients with poor prognosis (Reedijk *et al*, 2005). Despite these associations, until recently, the functional mechanism of Notch signalling in breast cancer metastasis was poorly defined.

## Notch signalling and bone metastasis

Corroborating previously published data, we found that elevated expression of Jagged1 was associated with an increased incidence of breast cancer relapse. These findings were extended by interrogating a separate clinical data set with more diverse outcome measures such as organ-specific metastasis, which showed that elevated Jagged1-expression levels was associated with bone metastasis. Functional studies in mice demonstrated that Jagged1-expressing breast cancer cells promote bone metastasis by activating the Notch signalling pathway in the bone microenvironment (Sethi *et al*, 2011) (Figure 1B). These findings suggest a new paradigm for Notch signalling in breast cancer progression, defining a requirement for the pathway in the bone stroma as opposed to tumour cells during the formation of bone metastasis. Interestingly, a role for the Notch pathway in the tumour-associated stroma has been shown to facilitate other cancer-promoting functions, such as angiogenesis. In head and neck squamous cell carcinoma, cancer-mediated activation of the pathway in endothelial cells supported tumour neovascularisation and angiogenesis (Zeng *et al*, 2005). Similarly, Delta-mediated Notch signalling was shown to facilitate tumour angiogenesis by maintaining the sprouting integrity of the developing tumour vasculature (Noguera-Troise *et al*, 2006).

In the bone microenvironment, tumour-derived Jagged1 engaged the Notch pathway in two distinct cell types: osteoblasts and osteoclasts. Jagged1-mediated activation of the pathway in osteoblasts conferred a growth advantage to bone metastatic tumour cells. Mechanistic studies revealed that the proliferative gain was dependent on osteoblast-secreted IL-6, which was transcriptionally regulated by the Notch pathway and its downstream target Hey1 (Sethi *et al*, 2011). Independent studies have shown that IL-6 is associated with a poor prognosis in breast cancer (Zhang and Adachi, 1999; Salgado *et al*, 2003) and is capable of supporting tumour growth in stromal-dependent mechanisms (Sasser *et al*, 2007; Studebaker *et al*, 2008; Ara *et al*, 2009). In neuroblastoma (Ara *et al*, 2009) and multiple myeloma (Mitsiades *et al*, 2006), IL-6 derived from stromal cells was shown to be an important mediator between cancer cells and the bone microenvironment by supporting tumour survival and affecting osteoclast differentiation, respectively. Of note, activation of other developmental pathways in osteoblasts has also been shown to promote bone metastasis (Sethi and Kang, 2011). Paracrine Sonic Hedgehog signalling by prostate cancer cells was shown to promote bone metastasis by inducing osteoblast differentiation through a Gli1-dependent mechanism (Zunich *et al*, 2009).

Jagged1-expressing tumour cells demonstrated a severe osteolytic phenotype in mice, suggesting a potential effect of tumour-derived Jagged1 on osteoclastogenesis (Sethi *et al*, 2011). Coculture experiments between Jagged1-expressing tumour cells and primary bone marrow cells demonstrated a strong induction of osteoclastogenesis. Often, tumour cells affect osteoclast maturation indirectly through osteoblast-dependent regulation of RANKL and OPG signalling (Lu *et al*, 2009); however, our results supported a distinct mechanism as Jagged1-expressing tumour cells directly associated with pre-osteoclasts to promote their maturation. These findings were supported by the direct application of recombinant Jagged1 protein to pre-osteoclasts, which demonstrated a strong upregulation of osteoclast maturation (Sethi *et al*, 2011). Transcriptional profiling of osteoclast-differentiation markers more definitively confirmed these results. Integrating these findings, it appears that tumour-derived Jagged1 directly engages Notch pathway in pre-osteoclasts, promoting their differentiation into mature, multinucleated osteoclasts, a mechanism that can help explain the severe osteolytic phenotype observed in Jagged1-mediated bone metastasis.

## Targeting molecular mediators of Notch-dependent bone metastasis

When metastatic cancer cells spread to the bone, the influence of the microenvironment and resultant adaptations undertaken by tumour cells alter them in ways that render them resistant to cell-autonomous therapies that would otherwise effectively treat their corresponding primary tumour (Emmenegger and Kerbel, 2010; Karlou *et al*, 2010). Experimental mouse models have also shown the therapeutic inadequacies of targeted agents in treating metastatic lesions (Man *et al*, 2002; Francia *et al*, 2009). These observations collectively support the rational for targeting the microenvironment of the metastatic lesion, in conjunction with the tumour cells directly, to better control disease that has spread to distant organs. The molecular mechanisms underlying Jagged1-mediated bone metastasis suggested that targeting the Notch pathway in the bone microenvironment might prove as an effective strategy in the treatment of bone metastasis. Currently, *γ*-secretase inhibitors (GSI) are small molecules that can potently disrupt the Notch pathway; they are also gaining popularity as anticancer agents based on preclinical studies (Shih Ie and Wang, 2007). Combining these pieces of information, we decided to test whether administration of GSI could interrupt communication between Jagged1-expressing tumour cells and the bone microenvironment in an attempt to treat breast cancer bone metastasis. GSI therapy reversed the bone metastasis-promoting functions of Jagged1 by disrupting the Notch pathway in bone stromal cells. Gene-expression analysis of bone metastases demonstrated that GSI-treated mice displayed a downregulation of Notch target genes in the tumour-associated stromal compartment, including a decrease in IL-6 levels. Overall, the results revealed a stromal-dependent mechanism for Notch signalling in supporting tumour outgrowth in the bone and suggest that targeting the pathway in the tumour-associated stroma may improve treatment for breast cancer bone metastasis (Sethi *et al*, 2011).

Importantly, other mediators and regulators contributed to Notch signalling in bone metastasis and therefore represent potential targets for therapeutic intervention. Jagged1 was shown to be the prime mediator of Notch-dependent bone metastasis and would be an ideal target for monoclonal antibody therapy as it is a cell-surface protein. Monoclonal antibodies targeting other Notch pathway ligands, such as Delta-like 4, have been shown to disrupt angiogenesis and tumour growth, demonstrating the potential therapeutic advantage of targeting Notch ligands (Noguera-Troise *et al*, 2006; Ridgway *et al*, 2006). The cytokine IL-6 is released by osteoblasts in response to Jagged1-mediated bone metastasis and a potential therapeutic target. Tocilizumab is a humanised monoclonal antibody against the interleukin-6 receptor (IL-6R) that has been approved to be used as an immunosuppressive drug in the treatment of rheumatoid arthritis. Additional inhibitors of IL-6 and its downstream Jak-Stat pathways are also under active clinical development.

Of pathological significance, the TGF-*β* pathway was shown to regulate Jagged1 expression during breast cancer bone metastasis. TGF-*β* is sequestered in the bone matrix and often times released in response to bone degradation, a process that is largely at play during osteolytic bone metastasis (Korpal *et al*, 2009). TGF-*β* was established as a critical regulator of Jagged1-mediated bone metastasis. Enforced expression of Jagged1 in SMAD4-knockdown breast cancer cells, which are severely impaired in their ability to form productive osteolytic bone metastases due to the defective reception of microenvironment TGF-*β* cues (Kang *et al*, 2003, 2005), restored the bone metastasis-promoting functions of these malignant cells. The TGF-*β* pathway has also been targeted by therapeutic agents currently being tested in clinical trials (Korpal and Kang, 2010) and would be a candidate pathway to disrupt in combination with Notch signalling in the treatment of breast cancer bone metastasis.

As we appreciate the therapeutic potential of molecular mediators that support communication between tumour cells and the bone microenvironment in the generation of bone metastasis, it will be critical to test the efficacy of these agents in the appropriate patient population. With respect to Notch signalling in bone metastasis, there is preliminary data that link Jagged1 and IL-6 expression to the basal-like subtype of breast cancer (Sansone *et al*, 2007; Sethi *et al*, 2011, unpublished observation). It is therefore imperative to design robust clinical trials, evaluating these agents in select patient groups that are most likely to benefit from the particular therapeutic intervention. These agents should be administered either alone or in combination with other inhibitors targeting the cross-talk between tumour and bone stromal cells in the treatment of bone metastasis.

## Figures and Tables

**Table 1:** Examples of tumour-intrinsic and extrinsic Notch signalling in cancer progression

**Tumour type**	**Mechanism of Notch activation/inhibition**	**Stage of cancer progression**	**References**
*Tumour-intrinsic activation*			
T-ALL	Translocation between chromosome 7 and 9 leading to truncated TAN-1 (Notch1) transcripts	Oncogenesis, tumour initiation	Ellisen *et al*, 1991
T-ALL	Activating mutations in an extracellular and the PEST domain of *NOTCH1* found in greater than 50% of patients	Oncogenesis, tumour initiation	Weng *et al*, 2004
Breast cancer	MMTV studies and transgenic mouse models demonstrating that a truncated form of Int-3 (Notch4) is oncogenic	Oncogenesis, tumour initiation	Gallahan & Callahan, 1987; Jhappan *et al*, 1992; Raafat *et al*, 2004
Breast cancer	MMTV studies showing that truncated forms of Notch1 is oncogenic	Oncogenesis, tumour initiation	Dievart *et al*, 1999
Breast cancer	Loss of Numb, a negative regulator of Notch signalling, is associated with breast cancer; Notch intracellular domain transforms breast epithelial cells	Oncogenesis, tumour initiation	Stylianou *et al*, 2006; Weijzen *et al*, 2002; Zang *et al*, 2007
Pancreatic cancer	Ligand-dependent activation of the Notch pathway	Oncogenesis	Mullendore *et al*, 2009
Colorectal cancer	Ligand-dependent activation of the Notch pathway	Oncogenesis	Rodilla *et al*, 2009
Pancreatic cancer	Genetic inhibition of Notch1 reduces NF-κB, VEGF and MMP-9 expression	Invasion	Wang *et al*, 2006
Breast cancer	Ligand-dependent and Notch intracellular domain-dependent activation of the Notch pathway	Proliferation, anoikis, EMT	Leong *et al*, 2007; Zavadil *et al*, 2004
Melanoma	Notch intracellular domain mediated activation of the Notch pathway	Oncogenesis, proliferation, metastasis	Balint *et al*, 2005
			
*Tumour-extrinsic activation*			
Squamous cell carcinoma	Ligand-dependent activation in endothelial cells; suppression using genetic and pharmacological approach	Angiogenesis	Zeng *et al*, 2005
Glioma, lung carcinoma	Ligand-dependent activation of the pathway; inhibition using polyclonal antibodies against Delta-like 4	Angiogenesis	Noguera-Troise *et al*, 2006; Ridgway *et al*, 2006
Breast cancer	Ligand-dependent activation in bone microenvironment, osteoblasts, and osteoclasts; suppression using genetic and pharmacological approach	Bone metastasis	Sethi *et al*, 2011

Abbreviations: EMT=epithelial-to-mesenchymal transition; MMP-9=matrix metalloproteinase-9; MMTV= mouse mammary tumour virus; T-ALL=T-cell acute lymphoblastic leukaemia; VEGF=vascular endothelial growth factor.
